# Inhibition of tiRNA-Gly-GCC ameliorates neointimal formation *via* CBX3-mediated VSMCs phenotypic switching

**DOI:** 10.3389/fcvm.2023.1030635

**Published:** 2023-02-03

**Authors:** Zhihua Rong, Fengshi Li, Rui Zhang, Shuai Niu, Xiao Di, Leng Ni, Changwei Liu

**Affiliations:** Department of Vascular Surgery, Peking Union Medical College Hospital, Chinese Academy of Medical Sciences and Peking Union Medical College, Beijing, China

**Keywords:** VSMC, phenotypic switching, neointimal formation, tRFs, tiRNA

## Abstract

**Background and aim:**

tRNA-derived fragments (tRFs) are a new class of non-coding RNAs involved in a variety of pathological processes, but their biological functions and mechanisms in human aortic smooth muscle cells (HASMCs) phenotype transition and vascular intimal hyperplasia are unclear.

**Methods/results:**

tiRNA-Gly-GCC is upregulated in synthetic HASMCs, atherosclerotic arteries, plasma, and the balloon injured carotid artery of rats. Functionally, the inhibition of tiRNA-Gly-GCC represses HASMCs proliferation, migration, and reversed dedifferentiation, whereas the overexpression of tiRNA- Gly-GCC have contrary effects. Mechanistically, tiRNA-Gly-GCC performs these functions on HASMCs *via* downregulating chromobox protein homolog 3 (CBX3). Finally, the inhibition of tiRNA-Gly-GCC could ameliorate neointimal formation after vascular injury *in vivo*.

**Conclusions:**

tiRNA-Gly-GCC is a mediator of HASMCs phenotypic switching by targeting CBX3 and inhibition of tiRNA-Gly-GCC suppresses neointimal formation.

## Graphical Abstract

tiRNA-Gly-GCC produced under stress situation act as vascular smooth muscle cells (VSMCs) phenotypic switching regulator *via* targeting CBX3 through a post-transcriptional mechanism that leading to VSMCs transition from contractile to synthetic phenotype and causing neointimal formation. 
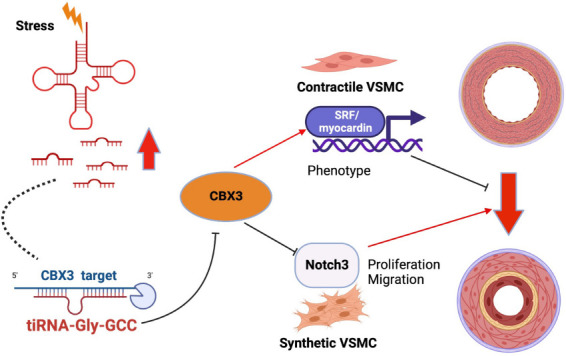

## 1. Introduction

Vascular smooth muscle cells (VSMCs) are the main component of blood vessels and play an essential role in vascular diseases, including atherosclerosis and restenosis after vascular angioplasty (1, 2). As non-terminally differentiated cells, VSMCs have strong plasticity (3, 4). Upon vascular injury, VSMCs change from the contractile phenotype in the physiological state to the synthetic phenotype in the activated state. During this transition, the proliferation and migration abilities of VSMCs are increased, accompanied by a decrease in VSMC-specific contractile genes, such as smooth muscle myosin heavy chain (SMMHC), smooth muscle α-actin (α-SMA), and smooth muscle calponin 1 (CNN1), and an increase in synthetic marker genes, such as Krueppel-like factor 4 (KLF4) (5). Activated VSMCs proliferate and migrate excessively into the vascular lumen to form neointima and lead to lumen stenosis (6). Currently, the specific mechanism leading to the phenotypic switching of VSMCs is still unclear. Therefore, it is necessary to investigate the mechanism of VSMCs phenotypic transformation.

During VSMCs phenotypic transition, non-coding RNAs (ncRNAs) play an important role (7, 8), such as miRNAs (5, 9, 10), lncRNAs (11–13), and circRNAs (14, 15). Among previously discovered ncRNAs involved in the phenotypic transition of VSMCs, here we propose that tRNA-derived fragments (tRFs), a new class of ncRNAs, generated by the specific cleavage of mature tRNAs or pre-tRNAs under stress conditions or under normal physiological conditions (16) rather than random degradation (17). According to their cleavage sites, tRFs can be divided into six subtypes, including tRF-1, tRF-2, tRF-3, tRF-5, internal tRF (itRF), and tRNA halves (tiRNA), the latter of which can be further divided into 5′-tiRNA and 3′-tiRNA (18). Previous studies have clarified that tRFs are involved in a variety of diseases, such as tumorigenesis (19, 20), neurodegenerative diseases (21), and in resistance to viral infections (22). Several studies have pointed out that tRFs are multifunctional ncRNAs and are involved in the regulation of cell proliferation, migration, and phenotypic switching (23). For example, tRF-Val promotes gastric cancer cell proliferation by targeting the chaperone molecule EEF1A1 (24). AS-tDR-007333 enhances non-small cell lung cancer cell proliferation and migration *via* the HSPB1/MED29 and ELK4/MED29 axes (25). tRF-21 regulates malignant phenotypes of pancreatic ductal adenocarcinoma cells by binding to hnRNPL (20). However, a role of tRFs in VSMCs phenotypic switching remains unclear.

Chromobox protein homolog 3 (CBX3) is a multifunctional molecule that regulates gene expression at the transcriptional level (26, 27), and plays an essential role in cell differentiation (28). For example, inhibition of CBX3 promotes the differentiation of preadipocytes to adipocytes (29). CBX3 can promote neural progenitor cell differentiation from embryonic stem cells by binding to the promoters of differentiation-related genes (30). More importantly, previous studies have shown that CBX3 can promote stem cell transformation into VSMCs (31). A recent research has confirmed that CBX3 can inhibit intimal hyperplasia after vascular injury by maintaining the contractile phenotype of VSMCs, but how CBX3 is regulated remains unclear (32). In this study, we identified that CBX3 is at least partially regulated by tiRNA-Gly-GCC, which is upregulated during VSMCs phenotypic switching *in vitro* and *in vivo* and find that tiRNA-Gly-GCC promotes human aortic smooth muscle cells (HASMCs) proliferation, migration, and dedifferentiation by targeting CBX3.

## 2. Materials and methods

### 2.1. Cell culture

Primary HASMCs were purchased from ScienCell (Cat# 6110, USA) and cultured in Smooth Muscle Cell Medium (ScienCell, USA), which contained 2% fetal bovine serum (FBS) and 1% Smooth Muscle Cell Growth Supplement (SMGS), at 37°C with 5% CO_2_. Cells at passages 4–8 were used in all experiments. HASMCs were cultured in serum-free and SMGS-free medium for 24 h before treatment with (50 ng/ml) platelet-derived growth factor-BB (PDGF-BB; R&D Systems, USA).

### 2.2. Human tRF and tiRNA sequencing

tRNA-derived fragment and tiRNA sequencing was performed as previously described (25). Briefly, total RNA was extracted using TRIzol (Invitrogen) from three pairs of HASMCs with or without PDGF-BB (50 ng/ml) treatment for 24 h and quantified by a NanoDrop (Thermo Fisher Scientific, USA). tRNA-derived fragments (tRF and tiRNA) are heavily modified by RNA modifications that interfere with small RNA-seq library construction. RNA modifications need to be removed before library construction, which is the biggest difference from common small RNA sequencing. RNA modifications were removed by the rtStar™ tRF and tiRNA Pretreatment Kit (Arraystar, Rockville, MD, USA). A sequencing library specific for tRFs was constructed from the pretreated total RNA by the following steps: (1) 3′ and 5′-adapter ligations and m1A and m3C demethylation; (2) cDNA synthesis; (3) PCR amplification; and (4) to differentiate tRFs with full-length tRNA, sequencing libraries are size-selected for the RNA biotypes to be sequenced using an automated gel cutter. Size of 134–160 bp PCR-amplified fragments (corresponding to a 14–40 nt small RNA size range) were selected, while the full length tRNA is 76–90 nt. The libraries were denatured into single-stranded DNA molecules, captured on Illumina flow cells, amplified *in situ* as sequencing clusters and sequenced for 50 cycles on an Illumina NextSeq 500 system (Illumina, CA, USA) using a NeXTs 500/550 V2 kit (#FC-404-2005, Illumina, CA, USA). The sequencing data has been uploaded to the GEO database (GEO accession number GSE212737).

The abundance of tRF and tiRNA was evaluated using their sequencing counts and was normalized as counts per million of total aligned reads (CPM). Differentially expressed tRFs and tiRNAs analyses was performed with R package edgeR (33). Fold change (cut-off 1.5), *p*-value (cut-off 0.05) were used for screening differentially expressed tRFs and tiRNAs. The number of subtypes tRFs, which the CPM of the sample or the average CPM of the group is not less than 20, can be counted against tRNA isodecoders which share the same anticodon but have differences in their body sequence. The stacked bar chart is plotted with R barplot package.

### 2.3. RNA extraction and quantitative real-time PCR

Total RNA was extracted using TRIzol (Invitrogen) from vasculature, cells, or plasma according to the manufacturer’s protocol. tiRNA reverse transcription, cDNA synthesis, and quantitative real-time PCR (qRT-PCR) were performed as previously described (34). Briefly, the rtStar™ tRF and tiRNA Pretreatment Kit (Cat# AS-FS-005, Arraystar, Rockville, MD, USA) was used to remove RNA modifications, and the rtStar™ First-Strand cDNA Synthesis Kit (3′ and 5′ adaptor) (Cat# AS-FS-003, Arraystar, Rockville, MD, USA) was used to synthesize cDNA. For mRNA reverse transcription, cDNA was synthesized using ReverTra Ace^®^ qPCR RT Master Mix (TOYOBO, Japan). For qRT-PCR, THUNDERBIRD^®^ Next SYBR qPCR Mix (TOYOBO, Japan) was used according to the manufacturer’s instructions, and the procedure was performed on a QuantStudio™ 5 Real-time PCR System (Applied Biosystems, USA). U6 and glyceraldehyde phosphate dehydrogenase (GAPDH) were regarded as the reference for tiRNA and mRNA, respectively. The expression levels were analyzed by the ΔC_q_ method, and 2^–ΔΔCq^ was used to calculate the relative expression. Primers are listed in Supplementary Table 1.

### 2.4. Western blot

Total protein of cells and vascular tissues was extracted with protein lysis buffer (Beyotime, China) and protease inhibitor mix (Thermo Fisher Scientific, USA). A BCA kit (Thermo Fisher Scientific, USA) was used for protein quantification. The protein extract denatured at 98°C was then separated by 10% SDS-PAGE, transferred to a 0.4 μm pore size PVDF membrane and blocked in a 5% skim milk solution for 1 h at room temperature. After washing in TBST solution, the membranes were incubated overnight at 4°C in the following antibody solutions: anti-α-SMA (1:10,000; Proteintech, Cat# 55135-1-AP, China), anti-SMMHC (1:1,000; Proteintech, Cat# 21404-1AP), anti-CNN1 (1:1,000; Cell Signaling Technology, Cat# 17819, USA), anti-PCNA (1:10,000; Abcam, Cat# ab92552, USA), anti-CBX3 (1:1,000; Proteintech, Cat #11650-2-AP), and anti-β-actin (1:1,000; Proteintech, Cat# 66009-1-Ig). On the following day, the membranes were washed and incubated in the corresponding secondary antibody solutions (1:10,000, EasyBio, China, Cat# BE0132-100) for 1 h at room temperature. An ECL Chemiluminescence Kit (Millipore, USA) was used to detect the protein signal. ImageJ was used for gray value analysis.

### 2.5. Cell transfection and infection

To overexpress or knockdown tiRNA-Gly-GCC *in vitro*, tiRNA-Gly-GCC mimics (100 nmol) and inhibitor (100 nmol) (Genepharma, China) were transfected into HASMCs with Lipofectamine 3000 (Thermo Fisher Scientific, USA) according to the manufacturer’s instructions. Negative control (NC, 100 nmol) and inhibitor negative control (inhibitor NC, 100 nmol) (Genepharma) were used as control groups. Sequences of the above-listed oligos are listed in Supplementary Table 2.

Adenoviral vectors expressing CBX3 (Ad-CBX3) (Hanbio Biotechnology, China) were used to overexpress CBX3, and green fluorescent protein (Ad-GFP) (Hanbio Biotechnology) as the control group at 100 multiplicities of infection (MOI) and then treated or not with PDGF-BB (50 ng/ml) for another 24 h *in vitro*.

*In vitro* cell experiments, all data were collected from at least three independent replicates.

### 2.6. Dual-luciferase reporter assay

The binding sites of tiRNA-Gly-GCC and CBX3 was predicted by the TargetScan^1^ and miRanda^2^ database. Luciferase reporter plasmids of CBX3 wild-type (CBX3-wt) and CBX3 mut-type (CBX3-mut) were designed and produced by Genepharma. Cotransfection of luciferase reporter plasmids with tiRNA-Gly-GCC mimics or NC was performed using Lipofectamine 3000 (Thermo Fisher Scientific, USA) in HASMCs. Sequences of tiRNA-Gly-GCC mimics and NC are listed in Supplementary Table 2. Twenty-four hours later, firefly luciferase activity was measured by a microplate reader (Bio-Tek, USA) using the Dual-Luciferase^®^ Reporter Assay System (Promega, USA), and renilla luciferase activity was used as an endogenous reference.

### 2.7. Cell proliferation and migration

CCK-8 kit (Dojindo, Japan) and Cell-Light EdU Apollo 488 *In Vitro* Kit (RiboBio, China) were used to detect HASMCs proliferation following their protocols. Briefly, for CCK-8, 8 × 10^3^ treated cells/well were seeded in 96-well plates and incubated for 24 h, and then 10 μl CCK-8 solution was added for 2 h at 37°C. The absorbance at 450 nm was measured using a microplate reader (Bio-Tek, USA). For the EdU assay, 1 × 10^4^ treated cells were incubated for 24 h in 96-well plates, and then, the culture media was replaced with 100 μl media containing 50 μm EdU for 2 h. Proliferating cells were stained green and were visualized under a fluorescence microscope (Nikon, Japan).

Human aortic smooth muscle cells migration was detected using the scratch test and Transwell assay. Briefly, scratch model Culture-Inserts (ibidi GmbH, Germany) were used for scratch experiments. A total of 7 × 10^3^ treated cells were seeded in the wells of Culture-Inserts overnight, the inserts were removed the next day, the culture medium was replaced with serum-free medium for 24 h, and photographs were taken every 24 h. For the Transwell assay, chambers (24-well plate, 0.8-mm pores, Corning, USA) were used. A total of 2 × 10^4^ treated cells suspended without FBS were seeded in the upper chambers, while 600 μl medium containing 20% FBS was added to the lower chambers. After culturing for 24 h, the non-migrated cells in the upper side of the chamber were wiped off with a cotton swab, and the migrated cells on the lower side were stained with 0.1% crystal violet (Beyotime). Pictures were taken from five randomly selected fields and analyzed with ImageJ.

### 2.8. Human sample collection

In this study, four pairs of arterial tissues from healthy adults and patients with atherosclerosis were collected at Peking Union Medical College Hospital (China) between October 2021 and July 2022. Healthy femoral artery samples were collected from patients requiring amputation due to trauma and no history of peripheral arterial disease. Diseased femoral arteries were collected from patients who underwent bypass grafting or amputation because of lower limb arteriosclerosis obliterans. Information about the patients whose vascular tissues were collected is provided in Supplementary Table 3.

Blood samples (4 ml) from 16 pairs of healthy adults and patients with atherosclerosis were collected into EDTA anticoagulant tubes. Plasma was collected by centrifugation at 3,000 × *g* for 15 min at 4°C and stored at −80°C. Normal plasma was obtained from routine physical examination samples, provided that the patients were not diagnosed with atherosclerosis. Atherosclerotic plasma samples were collected from patients who underwent carotid endarterectomy due to arteriosclerosis. Patients with hypertension, diabetes mellitus, cancer and acute infection were excluded. The characteristics of the patients are listed in Supplementary Table 4.

The processes involving human samples in this experiment were supported by the Ethics Review Committee of Peking Union Medical College Hospital (I-22PJ218). Patient consent was obtained before sample collection, and all experiments complied with the Declaration of Helsinki (35).

### 2.9. Animal experiments

SD rats from Vital River Laboratory Animal Technology Co. Ltd. (Beijing, China) were housed in a specific pathogen-free (SPF) barrier system with standard temperature and humidity room on 12-h light/dark cycles. Male SD rats with a body weight of 200–250 g were used for the carotid balloon injury model. Intraperitoneal injection of 1% pentobarbital was used to anesthetize the rats. The left carotid artery was separated, and a 2F balloon catheter (Edwards Lifesciences, USA) was inserted from the external carotid artery to the common carotid artery. After dilation with 0.2 ml of saline and rotationally withdrawal three times, the left external carotid artery was ligated. For tiRNA-Gly-GCC knockdown *in vivo*, antagomir (Genepharma) and pluronic gel (Sigma-Aldrich, USA) were used as described in previous studies (5). Briefly, after vascular injury, 0.1 ml of 30% pluronic gel containing diethyl pyrocarbonate (DEPC)-treated H_2_O (mock), 10 nmol antagomir negative control (antagomir-NC), or 10 nmol antagomir – tiRNA-Gly-GCC (antagomir-tiRNA-Gly-GCC) was applied perivascularly to injured carotid arteries. The contralateral carotid artery was not injured as in the sham group. Carotid arteries were collected for further experiments at 14 days after the operation.

All animal experiments were approved by the Animal Ethics Committee of Peking Union Medical College Hospital (No. XHDW-2019-001) and performed in accordance with National Research Council’s Guide for the Care and Use of Laboratory Animals.

### 2.10. HE and immunohistochemistry staining

Vascular tissues were collected and fixed overnight in 4% paraformaldehyde (Solarbio, China), dehydrated, and embedded in paraffin. Sections were cut to a thickness of 4 μm, and an HE automatic staining instrument (HistoCore SPECTRA ST, Leica, Germany) was used to perform the HE staining. Images were taken using a digital slice scanner (KFBIO, China). The degree of stenosis and the neointima/media ratio were calculated as previously described (36).

For tissue immunofluorescence staining, paraffin sections were dewaxed in xylene and antigen-retrieved in citrate (Solarbio). Sections were blocked in QuickBlock™ Blocking Buffer for Immunol Staining (Beyotime) for 1 h at room temperature and incubated with anti-α-SMA (1:200; Proteintech, Cat# 67735-1-Ig) and anti-PCNA (1:100; Proteintech, Cat# 60097-1-Ig) primary antibodies overnight at 4°C. Fluorescent secondary antibodies Alexa Flour 594-conjugated goat anti-rabbit IgG (1:200, Proteintech, Cat# SA00013-4) and Alexa Flour 488-conjugated goat anti-mouse (1:200, Proteintech, Cat# SA00013-1) were used to create fluorescent signals. Sections were observed with a laser confocal microscope (Nikon, AXR, Japan).

### 2.11. RNA-fluorescence *in situ* hybridization

RNA-fluorescence *in situ* hybridization (RNA-FISH) was performed as previously described (37) with minor modifications according to the instructions of RNA-FISH kits (Genepharma). Briefly, 4-μm paraffin sections were dewaxed, rehydrated, and digested with proteinase K at 37°C for 20 min. Sections were blocked in blocking solution for half an hour at room temperature. After denaturation at 78°C for 8 min, hybridization with the tiRNA-Gly-GCC probe (1 μmol, Genepharma) was performed at 37°C overnight. The next day, the sections were washed with 2× SSC solution. The fluorescent signal of tiRNA-Gly-GCC was amplified by the SA-Cy3-Biotin system (Genepharma). To observe the colocalization of tiRNA-Gly-GCC, VSMCs were labeled with the specific marker α-SMA (1:400, Proteintech). Immunofluorescence images were taken under a laser confocal microscope (Nikon, AXR, Japan).

The tiRNA-Gly-GCC probe sequence: CAGGCGAGAATTCT ACCACTGAACCACCCATGC.

### 2.12. Statistical analysis

Data were analyzed and presented as the mean ± SD using GraphPad Prism-9.3 (USA). Comparisons between two groups were performed using Student’s *t*-test. Comparisons between more than two groups were performed using one-way analysis of variance (ANOVA). At least three independent biological replicates were used for each experiment. *p* < 0.05 was considered statistically significant.

## 3. Results

### 3.1. Characteristics of tRF/tiRNA expression during HASMCs phenotype switching *in vitro*

**FIGURE 1:**
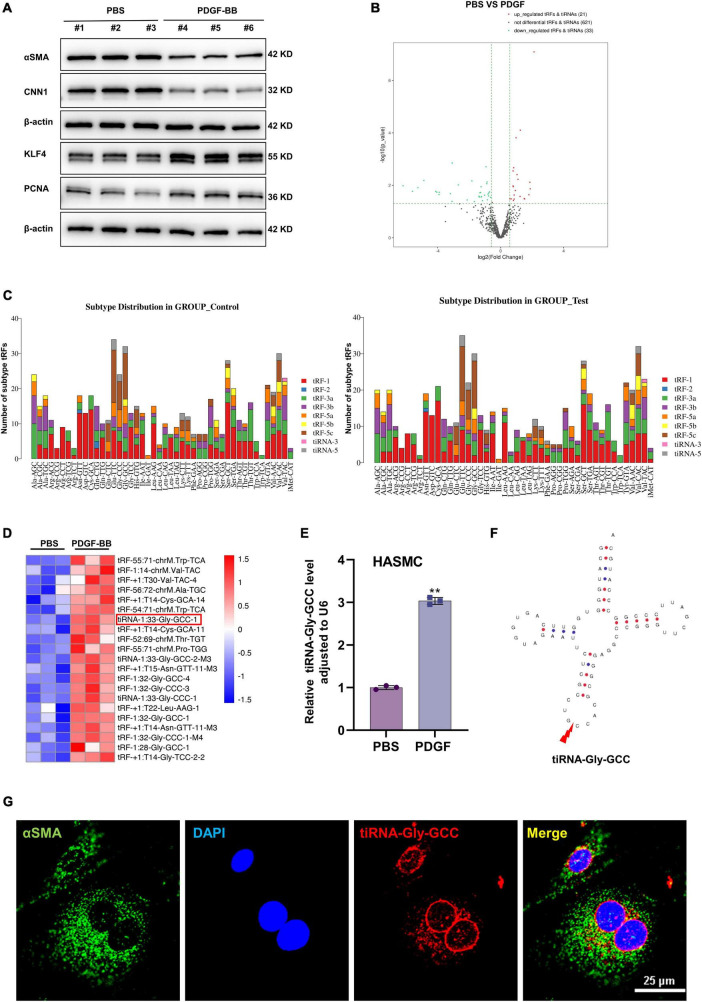
Characteristics of tRF profiles during human aortic smooth muscle cells (HASMCs) phenotypic switching *in vitro*. **(A)** Western blot analysis showing VSMC-specific proteins α-smooth muscle actin (α-SMA), calponin 1 (CNN1), synthetic protein Krueppel-like factor 4 (KLF4) and the cell proliferation marker, proliferating cell nuclear antigen (PCNA). Data were normalized to β-actin in HASMCs stimulated with platelet-derived growth factor-BB (PDGF-BB) (50 ng/ml). **(B)** Volcano plot showing differential expression profiles of tRFs in contractile (PBS) and synthetic (PDGF-BB) HASMCs, criteria for significant differences: | log2 (fold change) | >1 and *p* < 0.05. **(C)** Stacked plot for the number of tRFs derived from the same tRNA: the *X*-axis and *Y*-axis represent the tRNA names and the number of tRFs derived from this class of tRNA, respectively. tRFs are separated subtypes by their sites and length. **(D)** Heatmap of tRFs differentially expressed in contractile (PBS) and synthetic (PDGF-BB) HASMCs. **(E)** Quantitative real-time PCR (qRT-PCR) confirmed that the expression of tiRNA-Gly-GCC was increased in HASMCs stimulated with PDGF-BB (50 ng/ml) compared with PBS, normalized to U6. Data are presented as the mean ± SD. *n* = 3, **p < 0.01. **(F)** Structure of tRNA-Gly-GCC and the cleavage site of tiRNA-Gly-GCC. **(G)** RNA-fluorescence *in situ* hybridization (RNA-FISH) and cellular immunofluorescence staining confirmed that tiRNA-Gly-GCC (red, cy3 labeled) was partially colocalized with α-SMA (green, 488-labeled) in the cytoplasm of HASMCs, and 4′,6-diamidino-2-phenylindole (DAPI) (blue, labeled the nucleus). Scale bar, 25 μm.

Phenotypic switching of VSMCs is an important cause of vascular intimal hyperplasia, and PDGF-BB is an important stimulator of VSMCs phenotypic switching (14). Western blotting experiments showed that PDGF-BB (50 ng/ml) stimulation resulted in the downregulation of contractile proteins, including α-SMA and CNN1, and upregulation of the synthetic protein KLF4 and cell proliferation protein (proliferating cell nuclear antigen, PCNA), indicating that phenotypic switching of HASMCs *in vitro* was successful (Figure 1A). To explore the role of tRFs during VSMCs phenotypic switching, we performed tRF and tiRNA sequencing in contractile and synthetic HASMCs. The results of sequencing indicated that upon PDGF-BB stimulation (synthetic HASMCs), 21 tRFs were upregulated, while 33 tRFs were downregulated compared with the control group (contractile HASMCs) (Figure 1B). The subtype distribution demonstrated that tRFs derived from tRNA-Glu-TTC, tRNA-Gly-GCC, and tRNA-Val-CAC were the most abundant (Figure 1C). As showing in the heatmap of significantly upregulated (with a filter criteria of fold change >1.5 and *p* < 0.05) tRFs during HASMCs phenotypic switching, we selected tRFs with the most distinct expression differences and highest expression (Figure 1D and Supplementary Table 5) and verified them in HASMCs by qRT-PCR (Supplementary Figure 3). Among them, tiRNA-1:33-Gly-GCC-1 (hereafter referred to as tiRNA-Gly-GCC) have the highest abundance and expression (Figure 1E). tiRNA-Gly-GCC belonged to the subtype of tiRNA-5 and is cleaved from tRNA-Gly-GCC at site 33 (Figure 1F). RNA-FISH demonstrated that tiRNA-Gly-GCC and VSMC-specific protein α-SMA were partially colocalized in the cytoplasm of HASMCs (Figure 1G). The above results indicated that tRF/tiRNA expression is different during HASMCs phenotypic transition and that tiRNA-Gly-GCC is significantly upregulated in synthetic HASMCs, indicating that tiRNA-Gly-GCC may play an essential role during the transition process.

### 3.2. tiRNA-Gly-GCC was upregulated during VSMC phenotypic switching *in vivo*

**FIGURE 2:**
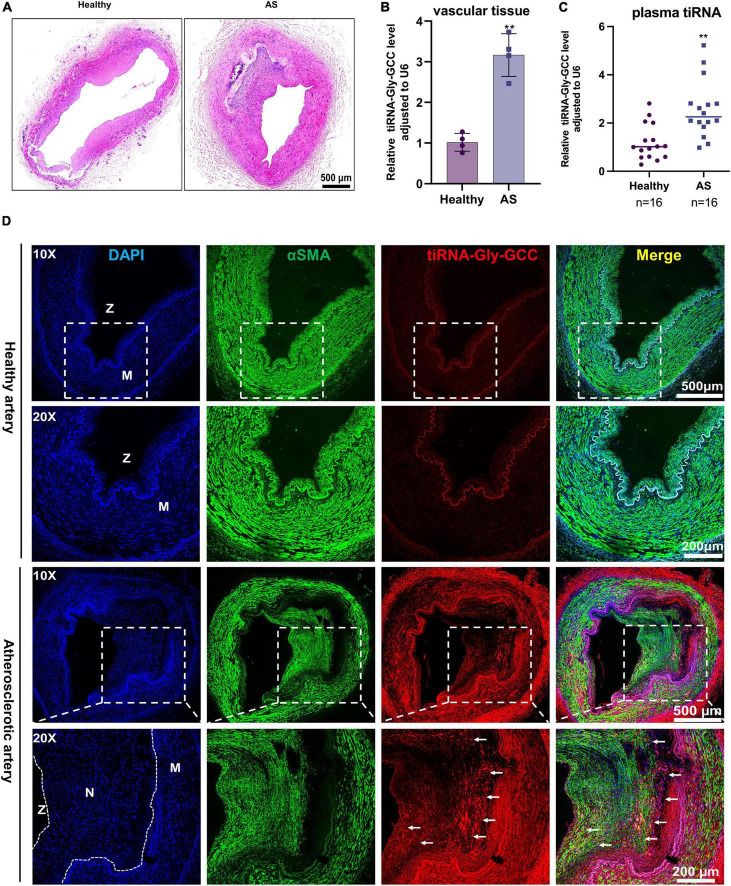
The tiRNA-Gly-GCC expression pattern in human atherosclerotic vascular tissues and plasma. **(A)** Representative HE staining of healthy and atherosclerotic arteries. *n* = 4; scale bar, 500 μm. **(B)** qRT-PCR analysis of tiRNA-Gly-GCC expression in healthy and atherosclerotic arteries (AS). Data are presented as the mean ± SD. *n* = 4, **p < 0.01. **(C)** tiRNA-Gly-GCC was analyzed by qRT-PCR in the plasma from healthy adults and patients with atherosclerosis, normalized to U6. Data are presented as the mean ± SD. *n* = 16 for each group, **p < 0.01. **(D)** Representative RNA-FISH and immunofluorescence staining of healthy and atherosclerotic arteries were selected from three group figures. tiRNA-Gly-GCC (red, cy3 labeled); α-SMA (green, 488-labeled); and DAPI (blue, labeled nucleus). The upper panel refers to healthy and the lower panel to atherosclerotic arteries. Scale bar, 500, 200 μm. N, neointimal; M, media of vascular; Z, zoom of vascular.

**FIGURE 3:**
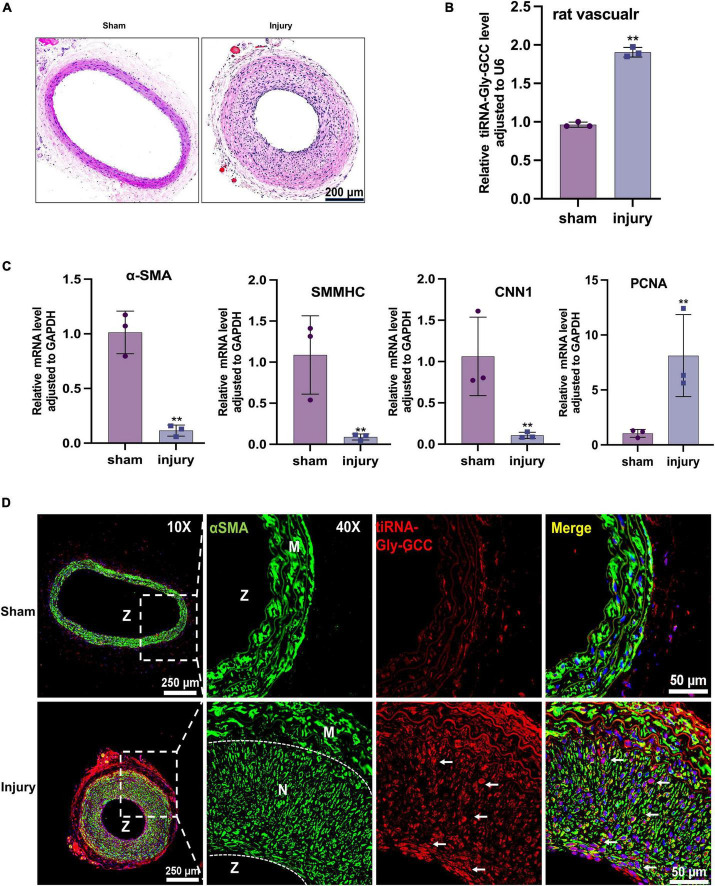
The tiRNA-Gly-GCC expression pattern in the rat vascular injury model. **(A)** Representative HE staining of sham and injured rat carotid arteries. *n* = 3; scale bar, 200 μm. **(B)** qRT-PCR showing the expression of tiRNA-Gly-GCC in sham and injured arteries. Data were normalized to U6. Data are presented as the mean ± SD. *n* = 3, **p < 0.01. **(C)** qRT-PCR showing the mRNA levels of VSMC-specific genes (α-SMA, SMMHC and CNN1) and PCNA in sham and injured arteries. Data were normalized to GAPDH. Data are presented as the mean ± SD. *n* = 3, **p < 0.01. **(D)** Representative RNA-FISH and immunofluorescence staining of sham and injured arteries. tiRNA-Gly-GCC (red, cy3 labeled); α-SMA (green, 488-labeled); and DAPI (blue, labeled nucleus). The upper panel refers to sham and the lower panel to injured arteries. *n* = 3, scale bar, 250, 50 μm. N, neointimal; M, vascular media; Z, zoom of vascular.

To investigate the role of tiRNA-Gly-GCC *in vivo*, we collected human vascular tissues and plasma from healthy adults and atherosclerotic patients. As shown in Figure 2A, atherosclerotic vessels are characterized by intimal hyperplasia and lumen stenosis. The qRT-PCR results indicated that tiRNA-Gly-GCC was significantly upregulated in atherosclerotic vascular tissues and plasma (Figures 2B, C). Similarly, in rat carotid artery balloon injury models (Figure 3A), tiRNA-Gly-GCC was upregulated after vascular injury (Figure 3B), accompanied by the downregulation of VSMC-specific genes (αSMA, SMMHC, and CNN1) and the upregulation of PCNA (Figure 3C). Furthermore, RNA-FISH of vascular tissues from humans and rats confirmed that tiRNA-Gly-GCC was mainly colocalized with α-SMA in neointimal tissues (Figures 2D, 3D and Supplementary Figures 1, 2). These data demonstrated that tiRNA-Gly-GCC is upregulated in atherosclerosis and injured vasculature.

### 3.3. tiRNA-Gly-GCC overexpression promoted HASMCs migration, proliferation, and dedifferentiation

**FIGURE 4:**
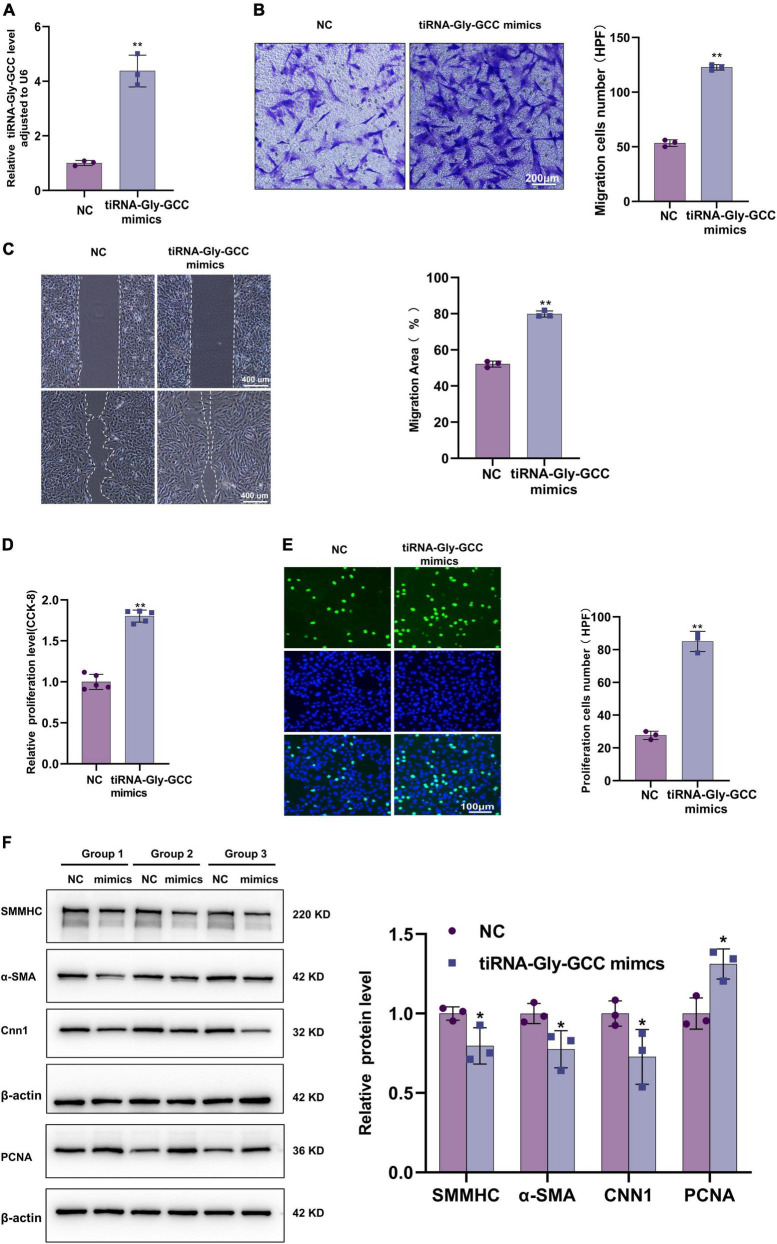
Overexpression of tiRNA-Gly-GCC promoted HASMCs proliferation, migration, and dedifferentiation. **(A)** qRT-PCR confirmed the overexpression efficiency of tiRNA-Gly-GCC mimics in HASMCs compared with negative control (NC), and data were normalized to U6. Data are presented as the mean ± SD. *n* = 3, **p < 0.01. **(B)** Transwell assays demonstrated that the overexpression of tiRNA-Gly-GCC promoted HASMCs migration. The left panel refers to representative image of Transwell assays and the right panel refers to quantification results of migrated HASMCs, from three independent experiments. Scale bar, 200 μm. Data are presented as the mean ± SD. *n* = 3, **p < 0.01. **(C)** Wound-healing assay showing that the overexpression of tiRNA-Gly-GCC promoted HASMCs migration. The left panel refers to representative image of wound healing assays and the right panel refers to quantification results of migration area, from three independent experiments. Scale bar, 400 μm. Data are presented as the mean ± SD. *n* = 3, **p < 0.01. **(D)** CCK-8 assays indicated that overexpression of tiRNA-Gly-GCC promoted the proliferation of HASMCs. Data are presented as the mean ± SD. *n* = 5, **p < 0.01. **(E)** EdU assays showing that overexpression of tiRNA-Gly-GCC promoted HASMCs proliferation. The left panel refers to representative image of EdU assays and the right panel refers to quantification results of proliferating HASMCs, from three independent experiments. Scale bar, 100 μm. Data are presented as the mean ± SD. *n* = 3, **p < 0.01. **(F)** The left panel refers to representative image of Western blot analysis for the expression of SMMHC, α-SMA, CNN1, and PCNA on HASMCs transfected with negative control (NC) or tiRNA-Gly-GCC mimics. The right panel refers to densitometric quantification of these proteins expression normalized by β-actin, from three independent experiments. Data are presented as the mean ± SD. *n* = 3, *p < 0.05 (versus NC).

To further investigate the functions of tiRNA-Gly-GCC, overexpression of tiRNA-Gly-GCC was performed in HASMCs with synthetic mimics. The transfection efficiency was tested by qRT-PCR (Figure 4A). Transwell and wound healing experiments indicated that tiRNA-Gly-GCC promoted HASMCs migration (Figures 4B, C). The results of CCK-8 and EdU assays demonstrated that tiRNA-Gly-GCC accelerated HASMCs proliferation (Figures 4D, E). Phenotypic and proliferation-related proteins were measured using Western blotting, which revealed that tiRNA-Gly-GCC mimics downregulated the expression of contractile proteins, such as SMMHC, α-SMA, and CNN1, while they upregulated proliferation proteins, such as PCNA (Figure 4F). These data indicated that tiRNA-Gly-GCC promotes HASMCs migration, proliferation, and dedifferentiation *in vitro*.

### 3.4. Inhibition of tiRNA-Gly-GCC reduced HASMCs migration and proliferation and reversed the dedifferentiation induced by PDGF-BB

**FIGURE 5:**
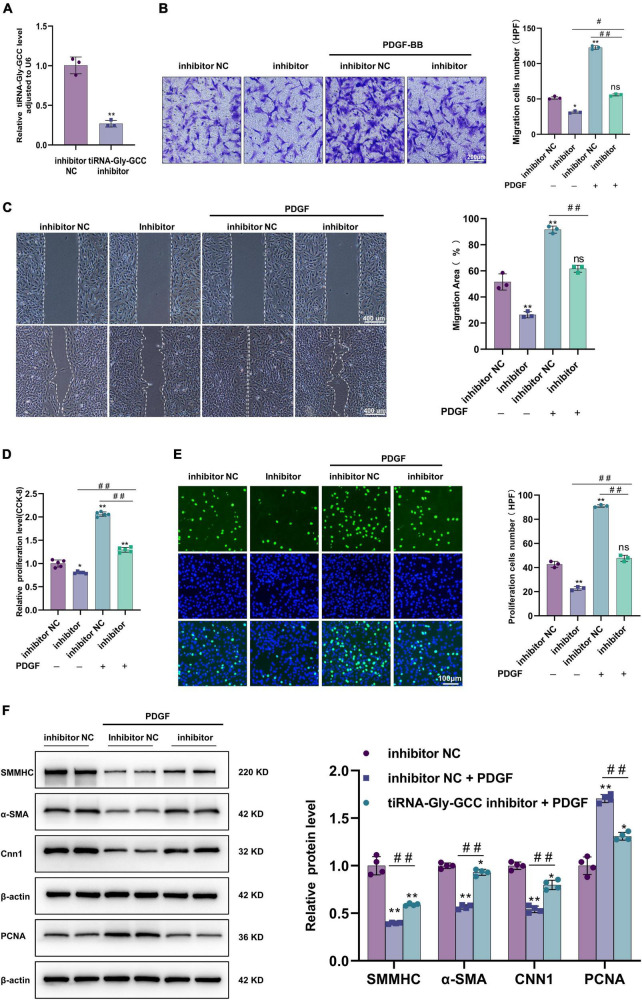
Inhibition of tiRNA-Gly-GCC suppressed HASMCs proliferation and migration and reversed the HASMCs dedifferentiation induced by PDGF-BB. **(A)** qRT-PCR confirmed the inhibition efficiency of the tiRNA-Gly-GCC inhibitor compared to inhibition negative control (inhibition NC) in HASMCs, normalized to U6. Data are presented as the mean ± SD. *n* = 3, **p < 0.01. **(B)** The left panel refers to representative image of Transwell assays and the right panel refers to quantification results of migrated HASMCs, from three independent experiments. HASMCs were transfected with inhibitor NC or tiRNA-Gly-GCC inhibitor then treated with PDGF-BB (50 ng/ml) or not. Data are presented as the mean ± SD. *n* = 3, *p < 0.05, **p < 0.01, ns: not significant (versus inhibitor NC). ^#^*p* < 0.05, ^##^*p* < 0.01 compared with the two groups, which are labeled by a horizontal line. **(C)** The left panel refers to representative image of wound healing assays and the right panel refers to quantification results of migration area, from three independent experiments. Scale bar, 400 μm. HASMCs were transfected with inhibitor NC or tiRNA-Gly-GCC inhibitor then treated with PDGF-BB (50 ng/ml) or not. Data are presented as the mean ± SD. *n* = 3, **p < 0.01, ns: not significant (versus inhibitor NC). ^#^*p* < 0.05, ^##^*p* < 0.01 compared with the two groups, which are labeled by a horizontal line. **(D)** CCK-8 assays indicated that the inhibition of tiRNA-Gly-GCC reduced the proliferation of HASMCs treated or not with PDGF-BB (50 ng/ml). Data are presented as the mean ± SD. *n* = 5, *p < 0.05, **p < 0.01 (versus inhibitor NC). ^##^*p* < 0.01 compared with the two groups, which are labeled by a horizontal line. **(E)** The left panel refers to representative image of EdU assays and the right panel refers to quantification results of migrating cells, from three independent experiments. HASMCs were transfected with inhibitor NC or tiRNA-Gly-GCC inhibitor then treated with PDGF-BB (50 ng/ml) or not. Data are presented as the mean ± SD. *n* = 3, **p < 0.01, ns: not significant (versus inhibitor NC). ^##^*p* < 0.01 compared with the two groups, which are labeled by a horizontal line. **(F)** The left panel refers to representative image of Western blot analysis for the expression of SMMHC, α-SMA, CNN1, and PCNA on HASMCs transfected with inhibitor NC or tiRNA-Gly-GCC inhibitor then treated with PDGF-BB or not (50 ng/ml). The right panel refers to densitometric quantification of these proteins expression normalized by β-actin, from three independent experiments. Data are presented as the mean ± SD. *n* = 3, *p < 0.05, **p < 0.01 (versus inhibitor NC). ^##^*p* < 0.01 compared with the two groups, which are labeled by a horizontal line.

As shown in Figure 5A, a chemically synthesized inhibitor downregulated the expression of tiRNA-Gly-GCC in HASMCs. Contrary to the results of tiRNA-Gly-GCC overexpression, Transwell and wound-healing experiments demonstrated that inhibition of tiRNA-Gly-GCC reduced the migration of HASMCs with or without PDGF-BB stimulation (Figures 5B, C). Similarly, the results of CCK-8 and EdU assays indicated that the inhibition of tiRNA-Gly-GCC retarded the migration of HASMCs induced by PDGF-BB compared with the control group (inhibitor NC) (Figures 5D, E). Furthermore, Western blotting revealed that the inhibition of tiRNA-Gly-GCC reversed the downregulation of contractile proteins (SMMHC, α-SMA, and CNN1) and the upregulation of PCNA induced by PDGF-BB *in vitro* (Figure 5F). Overall, inhibition of tiRNA-Gly-GCC reduces HASMCs proliferation and migration and reverses the dedifferentiation induced by PDGF-BB.

### 3.5. CBX3 is the target of tiRNA-Gly-GCC

**FIGURE 6:**
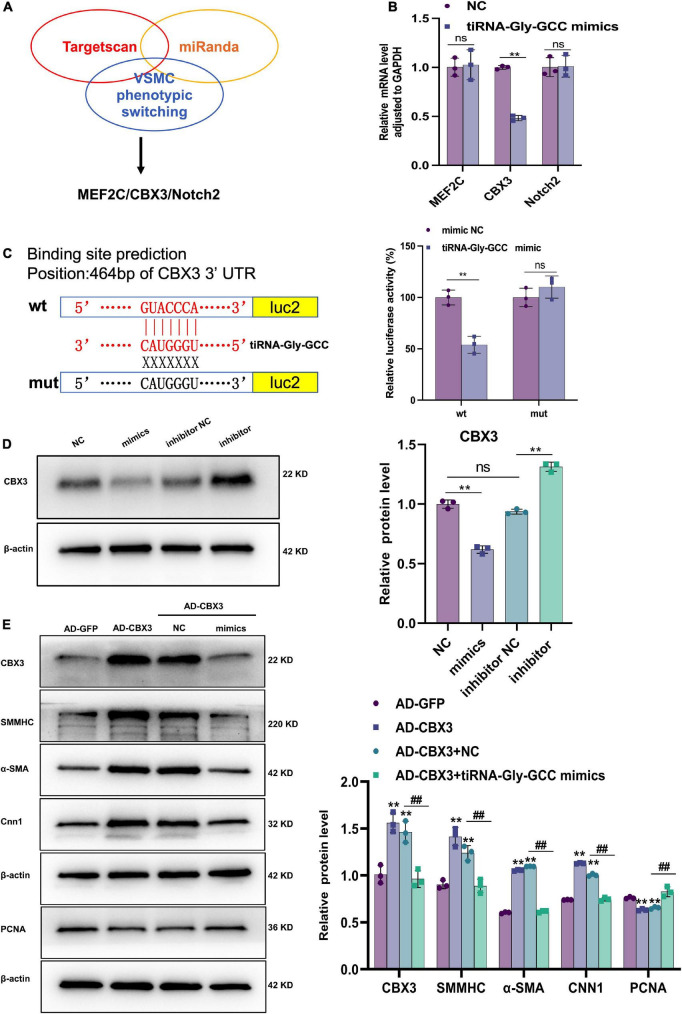
CBX3 is the target of tiRNA-Gly-GCC. **(A)** Targets of tiRNA-Gly-GCC were predicted by TargetScan and miRanda database. Then the targets involved in VSMCs phenotypic switching were selected. **(B)** The mRNA levels of potential target genes (MEF2C, CBX3, and Notch2) were analyzed using qRT-PCR after HASMCs were transfected with NC or mimics for 24 h and normalized to GAPDH. Data are presented as the mean ± SD. *n* = 3, **p < 0.01, ns: not significant (versus NC). **(C)** The right panel represents a schematic representation of dual-luciferase reporter gene assay about tiRNA-Gly-GCC binding sites within CBX3 3′UTR. Wild-type group (wt), mutant group (mut). Luc2, firefly luciferase reporter gene. The right panel refers to quantitative results of fluorescence intensity, from three independent experiments. Data are presented as the mean ± SD. *n* = 3, **p < 0.01, ns: not significant (versus mimic NC). **(D)** The left panel refers to representative image of Western blot analysis for the expression of CBX3 on HASMCs transfected with negative control (NC), tiRNA-Gly-GCC mimic, inhibitor NC or tiRNA-Gly-GCC inhibitor. The right panel refers to densitometric quantification of CBX3 expression normalized by β-actin, from three independent experiments. Data are presented as the mean ± SD. *n* = 3, **p < 0.01, ns: not significant, compared with the two groups, which are labeled by a horizontal line. **(E)** The left panel refers to representative image of Western blot analysis for the expression of CBX3, SMMHC, α-SMA, CNN1, and PCNA. The right panel refers to densitometric quantification of these proteins expression normalized by β-actin, from three independent experiments. HASMCs were infected with AD-GFP (control) or AD-CBX3 vector for 24 h and then transfected with NC or tiRNA-Gly-GCC mimics for another 24 h. Data are presented as the mean ± SD. *n* = 3, **p < 0.01 (versus AD-GFP). ^##^*p* < 0.01 compared with the two groups, which are labeled by a horizontal line.

The TargetScan and miRanda databases were used to predict the potential targets of tiRNA-Gly-GCC, among the potential target genes, MEF2C (38–41), CBX3 (31, 32), and Notch2 (42, 43) were involved in VSMCs phenotypic switching, proliferation and migration as previously described (Figure 6A and Supplementary Figure 4). The qRT-PCR results indicated that CBX3 mRNA levels were decreased only, while MEF2C and Notch2 were not significantly different when HASMCs were transfected with tiRNA-Gly-GCC mimics for 24 h (Figure 6B). Interestingly, the dual-luciferase reporter assay confirmed the binding sites of tiRNA-Gly-GCC within the CBX3 3′UTR (Figure 6C). Furthermore, the protein level of CBX3 was repressed in the tiRNA-Gly-GCC mimic group compared with NC group, while inhibition of tiRNA-Gly-GCC promoted the expression of CBX3 in HASMCs. To further confirm the regulatory mechanism, a rescue experiment was performed *in vitro*. As shown in Figure 6D, overexpression of CBX3 induced the expression of VSMC contractile proteins (SMMHC, α-SMA, and CNN1) while reducing the proliferation protein PCNA. However, tiRNA-Gly-GCC mimics reversed these effects of CBX3 (Figure 6E). The negative regulation of tiRNA-Gly-GCC with CBX3 and the results of the dual-luciferase reporter assay demonstrated that tiRNA-Gly-GCC regulates HASMCs phenotypic switching by targeting CBX3.

### 3.6. Inhibition of tiRNA-Gly-GCC *in vivo* ameliorates neointimal formation after vascular injury

**FIGURE 7:**
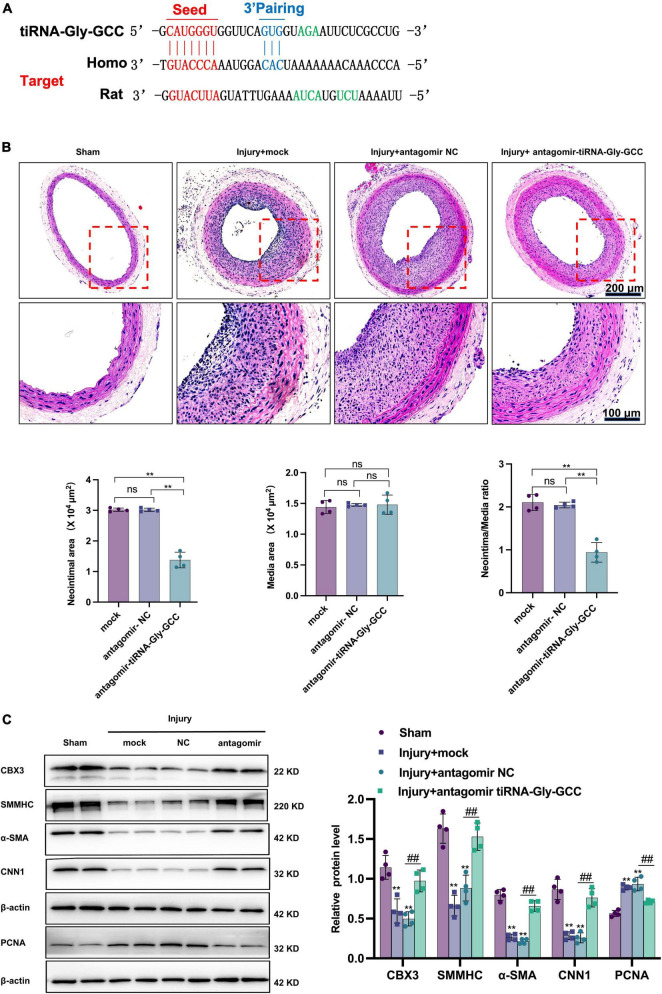
Inhibition of tiRNA-Gly-GCC reduced neointimal formation after vascular injury by targeting CBX3. **(A)** The binding sites of tiRNA-Gly-GCC within the CBX3 3′UTR of humans and rats. **(B)** The upper panel refers to representative HE staining of rat carotid arteries treated with PBS (mock) or antagomir NC or antagomir-tiRNA-Gly-GCC 14 days after injury. The lower panel refers to quantification results of neointimal area, media area, and neointimal/media ratio, from four independent experiments. Data are presented as the mean ± SD. *n* = 4, **p < 0.01, ns: not significant, compared with the two groups, which are labeled by a horizontal line. Scale bar, 200, 100 μm. **(C)** The left panel refers to representative image of Western blot analysis for the expression of CBX3, VSMC-specific proteins (SMMHC, α-SMA, and CNN1) and PCNA in the sham, mock, antagomir NC, and antagomir-tiRNA-Gly-GCC groups. The right panel refers to densitometric quantification of these proteins expression normalized by β-actin, from four independent experiments. Data are presented as the mean ± SD. *n* = 4 for each group, **p < 0.01 (versus sham group). ^##^*p* < 0.01 compared with the two groups, which are labeled by a horizontal line.

**FIGURE 8:**
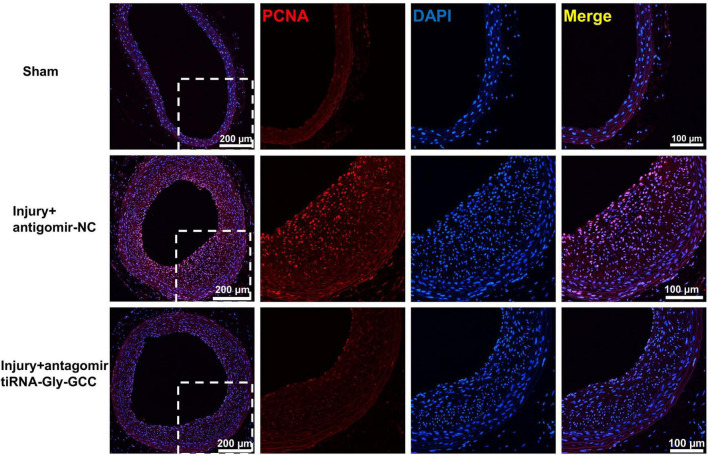
Inhibition of tiRNA-Gly-GCC reduced VSMC proliferation *in vivo*. Immunofluorescence staining demonstrated PCNA (red) and DAPI (blue) expression and localization in the sham group and injured arteries treated with antagomir NC or antagomir-tiRNA-Gly-GCC for 14 days, from four independent experiments. Scale bar, 200, 100 μm.

Since tiRNA-Gly-GCC exhibited a pronounced functional regulatory effect on HASMCs and its expression was upregulated after vascular injury in rats, we aimed to determine whether inhibition of tiRNA-Gly-GCC could ameliorate intimal hyperplasia after vascular injury in rats. As shown in Figure 7A, the predicted binding seed sites of tiRNA-Gly-GCC with CBX3 have a high degree of conservation in humans and rats. The inhibition of tiRNA-Gly-GCC *in vivo* significantly reduced neointimal formation without affecting the media area (Figure 7B). The Western blot results indicated that CBX3 and VSMCs contractile proteins (SMMHC, α-SMA, and CNN1) were significantly downregulated, while the proliferation-related protein PCNA was upregulated after vascular injury, but the inhibition of tiRNA-Gly-GCC *in vivo* upregulated the expression of these contractile proteins and downregulated the proliferation protein PCNA by increasing CBX3 (Figure 7C). Furthermore, tissue immunofluorescence staining confirmed that the inhibition of tiRNA-Gly-GCC significantly reduced the proliferation of VSMCs after vascular injury (Figure 8). These data demonstrated that inhibition of tiRNA-Gly-GCC can ameliorate neointimal formation by reducing VSMCs proliferation and dedifferentiation by targeting CBX3 *in vivo*.

## 4. Discussion

In this study, we identified that tiRNA-Gly-GCC is increased during HASMCs transition from a contractile to a synthetic phenotype by high-throughput sequencing. Moreover, it is confirmed that tiRNA-Gly-GCC is upregulated in the vascular tissues and plasma of atherosclerosis patients and balloon-injured rat carotid arteries. Overexpression of tiRNA-Gly-GCC *in vitro* promotes HASMCs proliferation and migration but represses the expression of VSMCs contractile proteins by decreasing CBX3. The inhibition of tiRNA-Gly-GCC results in the opposite effects and suppresses neointimal formation after vascular injury. These findings suggest that tiRNA-Gly-GCC is a potential therapeutic target for preventing restenosis after vascular injury and may be a promising biomarker for the diagnosis of atherosclerosis and restenosis.

Vascular intimal hyperplasia is a common pathological feature of atherosclerosis and restenosis after revascularization. The abnormal function of VSMCs is an important cellular event leading to vascular intimal hyperplasia. Upon vascular injury or stimulation with growth factors such as PDGF-BB, VSMCs change from a resting state to an active state. In this study, we innovatively proposed that tRFs, a new class of non-coding RNAs, were involved in the functional regulation of VSMCs, which is consistent with the latest studies. For example, 5′-tiRNA-Cys-GCA could reverse aortic dissection progression by suppressing VSMCs proliferation, migration and phenotypic switching by targeting STAT4 (44). Zhu et al. identified that tRF-Gln-CTG is upregulated upon vascular injury and prove its function in promoting VSMCs proliferation by suppressing the death receptor FAS (45).

Upon cellular stress, such as oxidative stress, hypoxia, and viral infection, tRNA could be cleaved at specific sites to form tRFs, and tiRNA is a special type that is cleaved specifically at the anticodon region of tRNA (46). Growing evidence suggests that tRFs are a novel type of gene regulator (47) that exhibit vital biological functions *via* the following mechanisms: (1) similar to miRNAs, tRFs interact with the 3′ untranslated region (3′UTR) of target mRNAs (48–51); (2) tRFs control the stability of mRNAs by binding to RNA-binding proteins (19, 20, 25); and (3) tRFs regulate protein translation by promoting ribosome biogenesis or interfering with translation initiation (52, 53). In this study, using bioinformatics analysis and molecular biology experiments, we confirmed that tiRNA-Gly-GCC promotes HASMCs proliferation and migration by targeting CBX3 in a manner like miRNAs. Consistent with this study, previous researchers have indicated that tiRNA-Gly-GCC is increased in papillary thyroid cancer (PTC) and promotes PTC cell proliferation and migration by targeting the RNA binding protein RBM17 (54). In human trophoblast cells, inhibition of tiRNA-Gly-GCC induces HTR8/SVneo apoptosis (55).

In this study, overexpression of CBX3 promotes HASMCs differentiation by upregulating contractile proteins, such as SMMHC, CNN1, and α-SMA. Our result is consistent with previous studies stating that CBX3 facilitates VSMCs differentiation from stem cells by regulating Dia-1 translocation and recruiting SRF to VSMCs contractile genes (31). Furthermore, CBX3 is downregulated after vascular injury, while the overexpression of CBX3 can suppress VSMCs proliferation, migration and neointimal formation by downregulating Notch3 (32). The results from this study confirmed that CBX3 is downregulated after vascular injury, while inhibition of tiRNA-Gly-GCC promotes the expression of CBX3, which results in the reduction of vascular intimal hyperplasia and suppression of the proliferation and migration abilities of HASMCs. In contrast, overexpression of tiRNA-Gly-GCC inhibits the expression of CBX3 and thus promotes the proliferation and migration of HASMCs. This negative relationship indicated that CBX3 is at least partially regulated by tiRNA-Gly-GCC in HASMCs.

Because tRFs are conserved (56), specific and expressed with great abundance in body fluids (57), they can be used as potential molecular diagnostic markers for cancer (25, 58), stroke (59), leukemia (60), aortic dissection (61), etc. In this study, we analyzed the expression of tiRNA-Gly-GCC in plasma from patients with or without atherosclerosis and found that tiRNA-Gly-GCC is significantly increased in atherosclerotic patients. Previous studies have indicated that tRF-Gly-GCC is upregulated in the plasma of atherosclerotic patients (62). These results imply that tiRNA-Gly-GCC might be a potential diagnostic marker of atherosclerosis or restenosis after vascular injury, which is our next field to explore.

There are some limitations in our study. First, the sequencing results revealed 21 upregulated and 33 downregulated tRFs during HASMCs phenotypic switching. In this study, we just focused on the upregulated genes, among the top ten differentially upregulated tRFs, tiRNA-Gly-GCC expression showed the most significant difference and had the highest abundance (Supplementary Table 5), so we selected it as the research target, but there were other differentially upregulated tRFs in the sequencing results, such as tRF-55:71-chrM.Trp-TCA, tRF-+1:T14-Gly-TCC-2-2 and the downregulated tRFs deserve further study, these will be our next research direction. Second, the clinical samples are limited in this study, and increasing the number of samples can increase the confidence of the results.

## 5. Conclusion

In summary, the study found that tiRNA-Gly-GCC is increased during HASMCs phenotypic transition *in vivo* and *vitro* and that inhibition of tiRNA-Gly-GCC could maintain the contractile phenotype, suppresses the proliferation and migration of HASMCs and reduces neointimal formation after vascular injury by targeting CBX3.

## Data availability statement

The datasets presented in this study can be found in a online repository. The names of the repository and accession number can be found below: https://www.ncbi.nlm.nih.gov/geo/, GSE212737.

## Ethics statement

The studies involving human participants were reviewed and approved by the Ethics Review Committee of Peking Union Medical College Hospital. The patients/participants provided their written informed consent to participate in this study. The animal study was reviewed and approved by the Animal Ethics Committee of Peking Union Medical College Hospital. Written informed consent was obtained from the owners for the participation of their animals in this study.

## Author contributions

ZR: methodology, investigation, validation, data curation, formal analysis, and writing—original draft. FL: methodology, investigation, validation, data curation, and formal analysis. RZ and SN: investigation, methodology, and data curation. XD: investigation and methodology. LN: conceptualization, project administration, formal analysis, writing—review and editing, and supervision. CL: conceptualization, formal analysis, data curation, writing—review and editing, funding acquisition, and supervision. All authors contributed to the article and approved the submitted version.

## Footnotes

1
https://www.targetscan.org/vert_72/

2
http://www.miranda.org/
