# Supplementary material for: Inhibition of tiRNA-Gly-GCC ameliorates neointimal formation via CBX3-mediated VSMCs phenotypic switching
Source: Front Cardiovasc Med. 2023 Feb 3;10:1030635. doi: 10.3389/fcvm.2023.1030635 (PMC9937027; doi:10.3389/fcvm.2023.1030635)
Supplement: Supplementary file 1 [file Data_Sheet_1.docx]

**Inhibition of tiRNA-Gly-GCC ameliorates neointimal formation *via* CBX3-mediated VSMCs phenotypic switching**

**Zhihua Rong^1#^, Fengshi Li^1#^, Rui Zhang^1^, Shuai Niu^1^, Xiao Di^1^, Leng Ni^1*^, Changwei Liu^1*^**

1 Department of Vascular Surgery, Peking Union Medical College Hospital, Chinese Academy of Medical Sciences and Peking Union Medical College, Beijing, 100730, People’s Republic of China.

**Supplementary Materials**

**Supplementary Figure 1**

**DAPI a-SMA Negative control Merge**


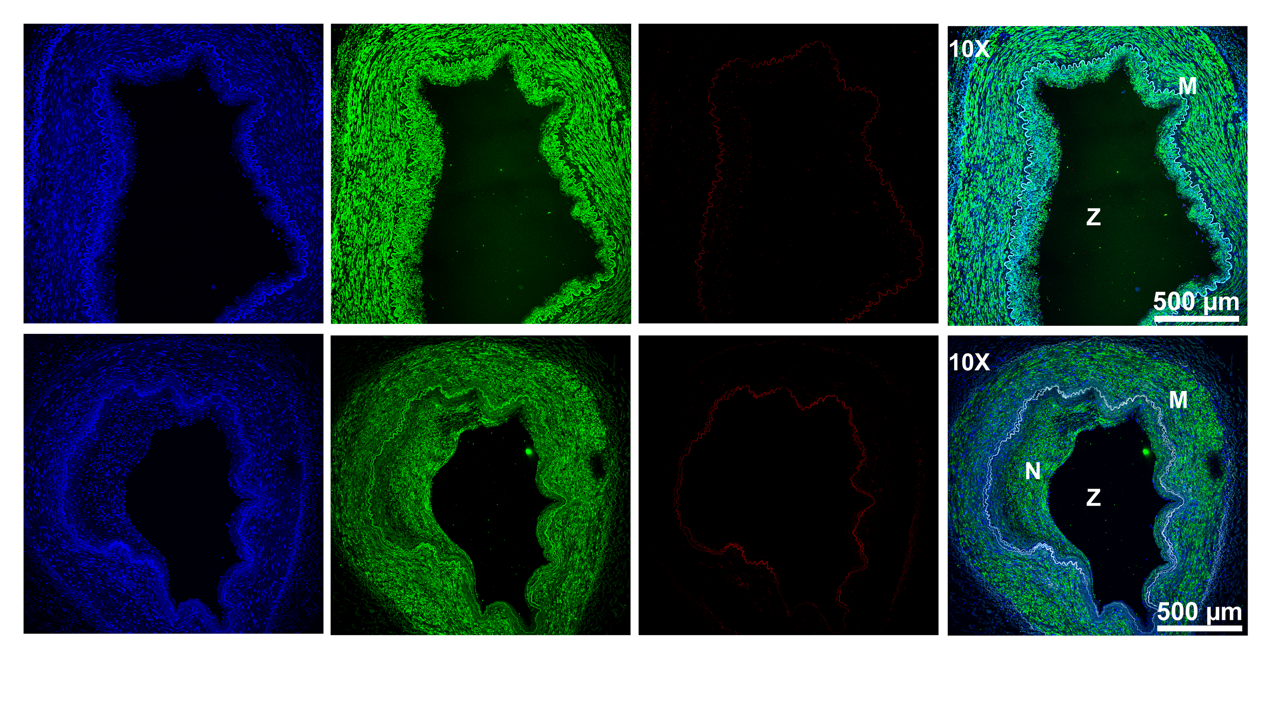


Representative RNA-FISH and immunofluorescence staining of healthy (upper) and atherosclerotic arteries (lower). Negative control probe (red, cy3 labelled); α-SMA (green, 488- labelled); DAPI (blue) labelled nucleus. Scale bar = 500 μm; N, neointimal; M, media of vascular; Z, zoom of vascular.

**Supplementary Figure 2**


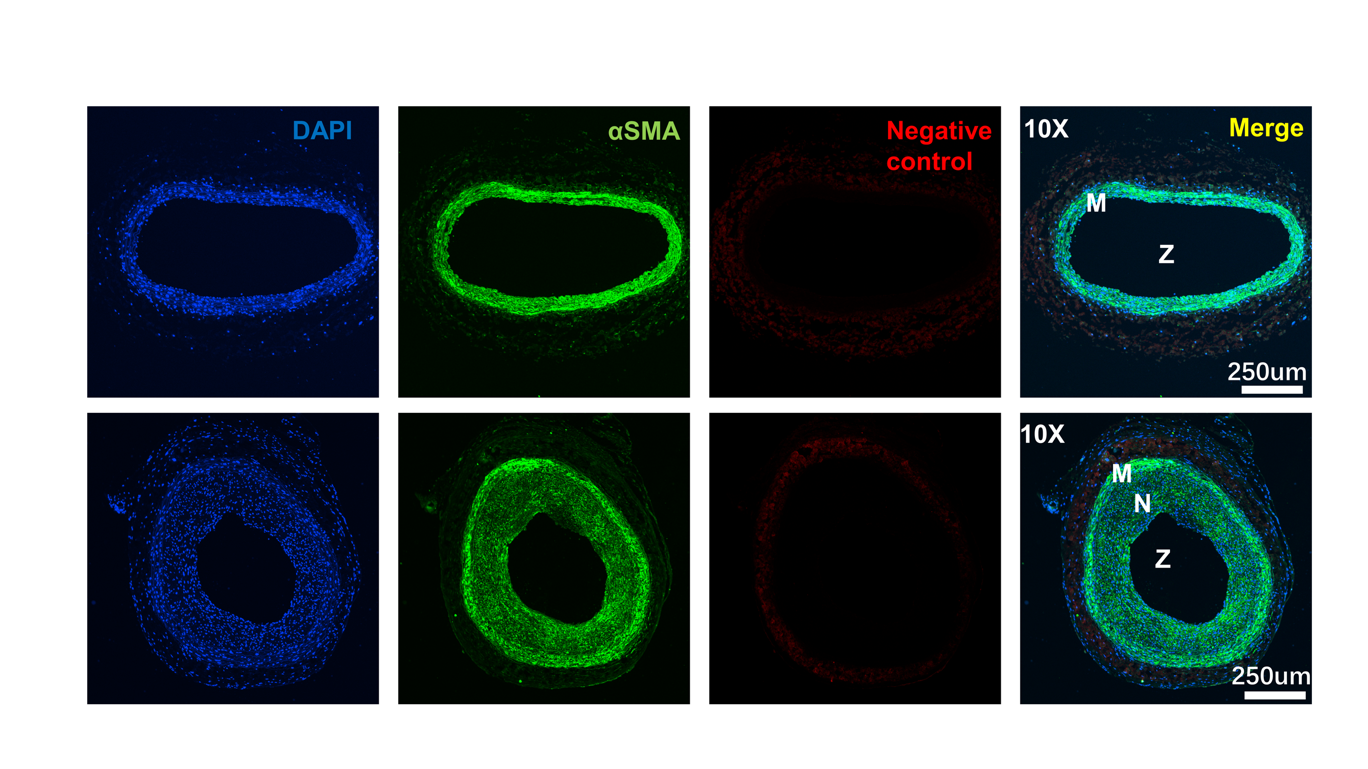


Representative RNA-FISH and immunofluorescence staining of sham (upper) and injured arteries (lower). Negative control probe (red, cy3 labelled); α-SMA (green, 488- labelled); DAPI (blue) labelled nucleus. Scale bar = 250 μm. N, neointimal; M, vascular media; Z, magnified view of the vasculature.

**Supplementary Figure 3**


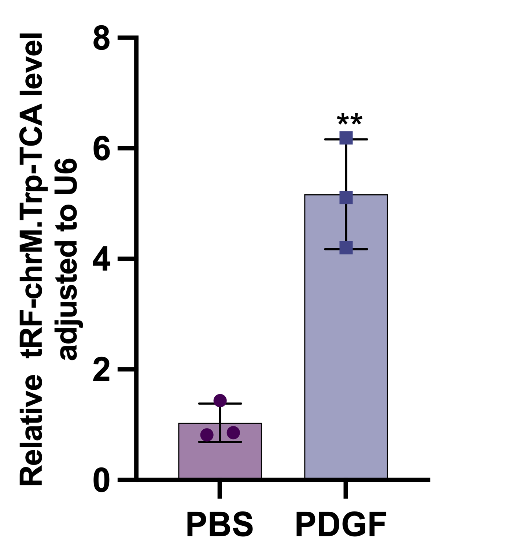

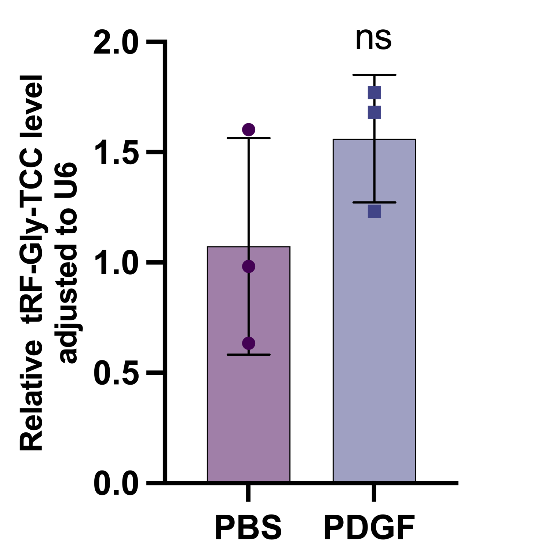


Quantitative real time PCR (qRT-PCR) confirmed that the expression of tRF-chrM.Trp-TCA (Right) and tRF-Gly-TCC (Left) normalized to U6 in HASMCs stimulated with PDGF-BB (50 ng/mL) compared with PBS, from 4 independent experiments. Data were presented as the mean ± SD. n = 3, ***p*<0.01

**Supplementary Figure 4**

**The targets of tiRNA-Gly-GCC were predicted by the TargetScan (**[**https://www.targetscan.org/vert_72/**](https://www.targetscan.org/vert_72/)**) and miRanda database (**[**http://www.miranda.org/**](http://www.miranda.org/)**).**

**The 2-7 and 13-16 nt of tRFs are called the seeding sequence and the 3’-pairing sequence, respectively. These two sequence pairings with mRNA are particularly important for site recognition.**


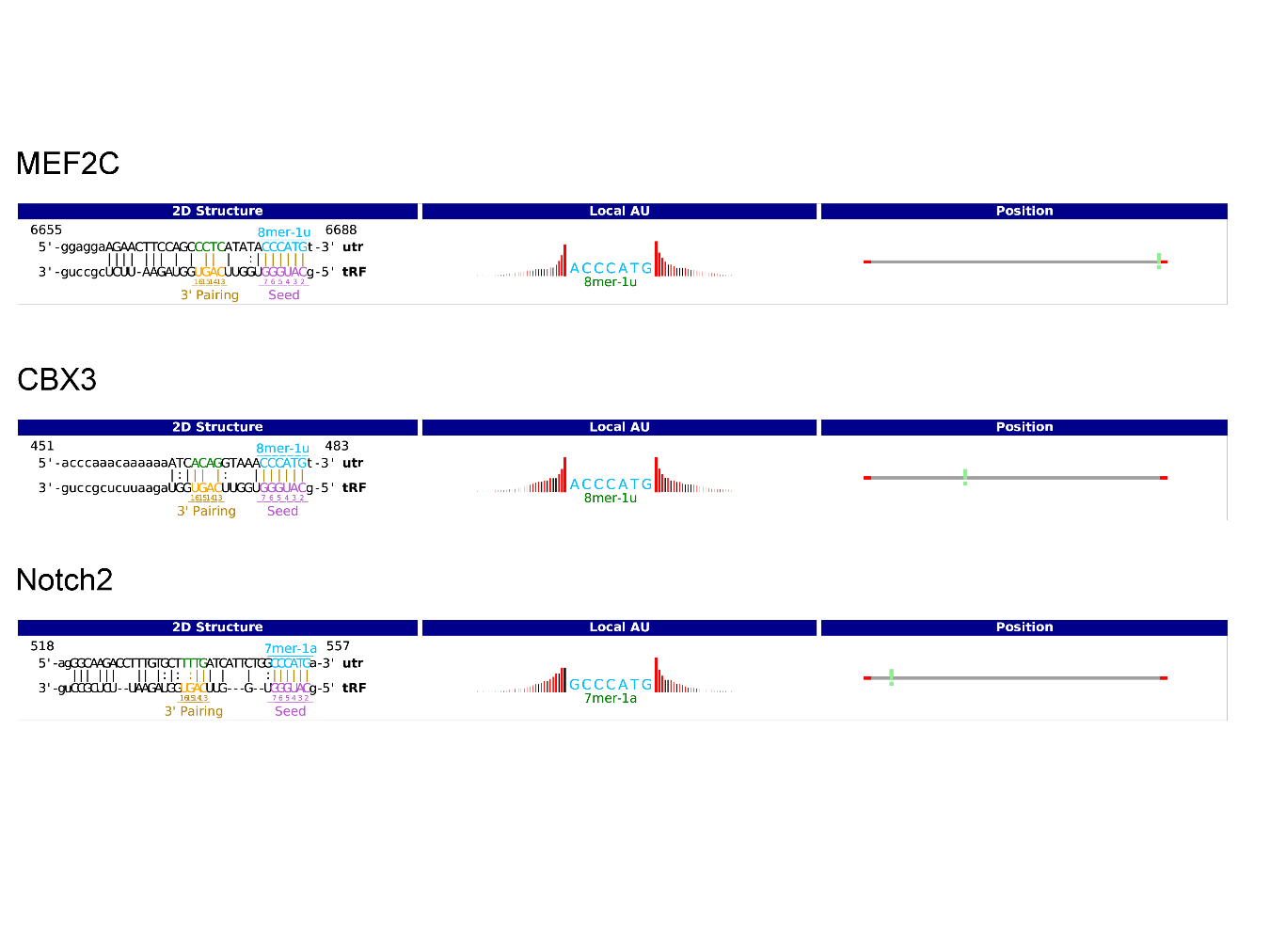


**Supplement Table 1:** PCR Primer Sequences

| **Name** | **sequences** |
| --- | --- |
| **RAT α-SMA forward primer**  **RAT α-SMA reverse primer**  **RAT SMMHC forward primer**  **RAT SMMHC reverse primer**  **RAT GAPDH forward primer**  **RAT GAPDH reverse primer**  **RAT PCNA forward primer**  **RAT PCNA reverse primer**  **RAT CNN1 forward primer**  **RAT CNN1 reverse primer**  **Homo GAPDH forward primer**  **Homo GAPDH reverse primer**  **Homo CBX3 forward primer**  **Homo CBX3 reverse primer**  **Homo** **MEF2C forward primer**  **Homo MEF2C reverse primer**  **Homo** **Notch2 forward primer**  **Homo Notch2 reverse primer**  **Homo U6 forward primer**  **Homo U6 reverse primer**  **tRF-chrM.Trp-TCA forward primer**  **tRF-chrM.Trp-TCA reverse primer**  **tiRNA-Gly-GCC-1 forward primer**  **tiRNA-Gly-GCC-1 reverse primer**  **tRF-Gly-TCC-2-2 forward primer**  **tRF -Gly-TCC-2-2 reverse primer** | **5’ CTGCCTTGGTGTGTGACAATGG 3’**  **5’ CGGGTACTTCAGGGTCAGGATTC 3’**  **5’ GCCGCTGCCTATGACAAACT 3’**  **5’ CGCTGGTTGTCCAAGTCCA 3’**  **5’ ACCCAGAAGACTGTGGATGG 3’**  **5’** **CACATTGGGGGTAGGAACAC 3’**  **5’ CCTGCTGGGACATCAGTTCG3’**  **5’ AAGCTGCACTAAGGAGACGTG3’**  **5’CGGCGTCACCTCTATGATCC3’**  **5’TCAAAGATCTGCCGCTTGGT3’**  **5’ ACAACTTTGGTATCGTGGAAGG 3’**  **5’ GCCATCACGCCACAGTTTC 3’**  **5’ GCCTGAAGAATTTGTCGTGGA 3’**  **5’ ACGCTTCAATCAATTCTGGACA 3’**  **5’ GCACCAACAAGCTGTTCCAG3’**  **5’ TGTCTGAGTTTGTCCGGCTC3’**  **5’ CCAGGCTATCAGGGTGTCAA3’**  **5’ ATGCCCTGGATGGAAAATGGAT3’**  **5’GCTTCGGCAGCACATATACTAAAAT3’**  **5’CGCTTCACGAATTTGCGTGTCAT3’**  **5’TCTACAGTCCGACGATCATACTTA 3'**  **5’TCTTCCGATCTTGGCAGAAA 3’**  **5’ TACAGTCCGACGATCGCATG 3'**  **5’ CGTGTGCTCTTCCGATCTCA 3’**  **5’ TTCTACAGTCCGACGATCGC 3'**  **5’ CTTCCGATCTAAAGGTCCGC 3’** |

**Supplemental Table 2:** The sequence of oligos

| NC | Sense UUCUCCGAACGUGUCACGUTT  Antisense ACGUGACACGUUCGGAGAATT |
| --- | --- |
| tiRNA-Gly-GCC mimics | Sense GCAUGGGUGGUUCAGUGGUAGAAUUCUCGCCUG  Antisense GGCGAGAAUUCUACCACUGAACCACCCAUGCUU |
| Inhibitor NC | CAGUACUUUUGUGUAGUACAA |
| tiRNA-Gly-GCC inhibitor | CAGGCGAGAAUUCUACCACUGAACCACCCAUGC |

**Supplemental Table 3:** Baseline Patient Characteristics (sample of vascular tissues)

| **Patient Characteristic** | **Healthy control group (n=4)** | **Atherosclerosis artery group (n=4)** | **P value** |
| --- | --- | --- | --- |
| **Demographics** | | | |
| Female sex，no.(%) | 2 (50%) | 1 (25%) | 0.465# |
| Age (years) | 40.5±4.8 | 72±5.6 | 0.569* |
| **Clinical Parameters** | | | |
| Smoker, no. (%) | 1 (25%) | 3 (75%) | 0.157# |
| BMI, kg/m2 | 26.83±0.9 | 31.47±1.55 | 0.179* |
| SBP, mmHg | 118.75±2.22 | 134.5±11.47 | 0.066* |
| DBP, mmHg | 80±2.16 | 87.25±2.75 | 0.379* |
| **Comorbidities** | | | |
| Carotid artery disease, no. (%) | 0 (0) | 3 (75%) | NA |
| Coronary artery disease, no. (%) | 0 (0) | 3 (75%) | NA |
| Hypertension, no. (%) | 0 (0) | 3 (75%) | NA |
| Type II diabetes mellitus, no. (%) | 0 (0) | 2 (50%) | NA |
| Hyperlipidemia, no. (%) | 0 (0) | 3 (75%) | NA |

N=4 *P* value is calculated with t-test (*), Fisher’s exact test (#). Carotid/coronary artery disease was defined as the arterial lumen stenosis rate greater than 70%. BMI, body mass index; SBP, systolic blood pressure; DBP, diastolic blood pressure. NA, not applicable.

**Supplemental Table 4:** Baseline Patient Characteristics (sample of plasma)

| **Patient Characteristic** | **Healthy control group (n=16)** | **Atherosclerosis group (n=16)** | | **P value** |
| --- | --- | --- | --- | --- |
| Age (years)  Gender (female)  BMI (Kg/m^2^)  Smoking (yes)  Heart rate (beats/min)  SBP (mmhg)  DBP (mmhg)  HDL (mmol/L)  LDL (mmol/L)  Total cholesterol (mmol/L)  Triglyceride (mmol/L) | 50.81±6.33  5  23.56±2.33  7  79.25±3.53  121.50±3.30  79.93±2.23  1.32±0.25  2.23±0.58  4.21±0.93  1.21±0.36 | | 52.87±3.74  4  24.18±2.34  8  77.18±4.18  124.31±4.12  79.93±4.98  1.07±0.34  2.68±0.88  4.45±1.48  1.23±0.39 | 0.271*  0.694#  0.625*  0.723#  0.142*  0.042*  1*  0.025*  0.104*  0.585*  0.894* |

N=16 *P* value is calculated with t-test (*), Fisher’s exact test (#). BMI, body mass index; SBP, systolic blood pressure; DBP, diastolic blood pressure; HDL, high-density lipoprotein; LDL, Low-density lipoprotein.

**Supplemental Table 5:**

| **tRF_ID** | **Type** | **Length** | **Fold**  **Change** | **P**  **Value** | **PBS**  **Average CPM** | **PDGF**  **Average**  **CPM** |
| --- | --- | --- | --- | --- | --- | --- |
| **tRF-55:71-chrM.Trp-TCA** | tRF-3a | 17 | 4.3548905 | 8E-08 | 28.44321 | 127.1978622 |
| tRF-1:14-chrM.Val-TAC | tRF-5a | 14 | 3.6793438 | 0.0137 | 4.839095 | 20.62218625 |
| tRF-+1:T30-Val-TAC-4 | tRF-1 | 30 | 3.6287743 | 0.0076 | 8.212952 | 29.87648378 |
| tRF-56:72-chrM.Ala-TGC | tRF-3a | 17 | 3.4781444 | 0.0229 | 5.895212 | 20.15020602 |
| tRF-+1:T14-Cys-GCA-14 | tRF-1 | 14 | 2.8770416 | 0.0332 | 7.37994 | 21.09523679 |
| tRF-54:71-chrM.Trp-TCA | tRF-3a | 18 | 2.8266035 | 0.0321 | 7.119631 | 20.62325656 |
| **tiRNA-1:33-Gly-GCC-1** | **tiRNA-5** | **33** | **2.3785753** | **8E-05** | 968.7348 | 2421.608623 |
| tRF-+1:T14-Cys-GCA-11 | tRF-1 | 14 | 2.3531736 | 0.0266 | 12.21998 | 31.64843575 |
| tRF-52:69-chrM.Thr-TGT | tRF-3a | 18 | 2.1484322 | 0.0059 | 35.09986 | 78.22349424 |
| tRF-55:71-chrM.Pro-TGG | tRF-3a | 17 | 2.0579676 | 0.0039 | 48.50611 | 102.2043928 |
| tiRNA-1:33-Gly-GCC-2-M3 | tiRNA-5 | 33 | 1.9724166 | 0.0002 | 4885.936 | 10024.96806 |
| tRF-+1:T15-Asn-GTT-11-M3 | tRF-1 | 15 | 1.9131432 | 0.0158 | 52.19708 | 107.2730568 |
| tRF-1:32-Gly-GCC-4 | tRF-5c | 32 | 1.8157702 | 0.0334 | 33.4134 | 64.24083195 |
| tRF-1:32-Gly-CCC-3 | tRF-5c | 32 | 1.8126739 | 0.0234 | 38.01168 | 71.83073824 |
| **tiRNA-1:33-Gly-CCC-1** | **tiRNA-5** | **33** | **1.782287** | **0.0021** | 1112.024 | 2057.060105 |
| tRF-+1:T22-Leu-AAG-1 | tRF-1 | 22 | 1.7551905 | 0.0375 | 184.6198 | 343.8990006 |
| **tRF-1:32-Gly-GCC-1** | **tRF-5c** | **32** | **1.7412489** | **0.0113** | 9693.512 | 17738.15542 |
| tRF-+1:T14-Asn-GTT-11-M3 | tRF-1 | 14 | 1.7310637 | 0.0106 | 120.4138 | 220.043338 |
| **tRF-1:32-Gly-CCC-1-M4** | **tRF-5c** | **32** | **1.7288622** | **0.0028** | 30332.09 | 51086.47616 |
| tRF-1:28-Gly-GCC-1 | tRF-5c | 28 | 1.5933387 | 0.0329 | 303.8658 | 506.5097908 |
| **tRF-+1:T14-Gly-TCC-2-2** | tRF-1 | 14 | 1.5033704 | 0.0328 | 6146.083 | 9665.562092 |

**The abundance of tRF & tiRNA is evaluated using their sequencing counts and is normalized as counts per million of total aligned reads [CPM]).** **tDRnamer(** [**http://trna.ucsc.edu/tDRnamer/**](http://trna.ucsc.edu/tDRnamer/) **) gtrna database (** [**http://gtrnadb.ucsc.edu**](http://gtrnadb.ucsc.edu) **) were used for the nomenclature used in tRF_ID and type.**
